# The therapeutic effect of genicular nerve radiofrequency for chronic knee pain after a total knee arthroplasty: A systematic review

**DOI:** 10.1016/j.inpm.2022.100072

**Published:** 2022-02-17

**Authors:** James B. Meiling, Brandon S. Barndt, Christopher T. Ha, James E. Eubanks, Justin B. Schappell, George M. Raum, Samir A. Khan, Larry Prokop, Aaron Conger, Zachary L. McCormick, Christine L. Hunt

**Affiliations:** a Department of Physical Medicine and Rehabilitation, Mayo Clinic, 200 First St SW, Rochester, MN, 55905, USA; b Department of Physical Medicine and Rehabilitation, Temple University Lewis Katz School of Medicine, Boyer Ste 226A, 3509 N Broad St, Philadelphia, PA, 19410, USA; c Department of Physical Medicine and Rehabilitation, University of Pittsburgh Medical Center, 1400 Locust St, Pittsburgh, PA, 15219, USA; d Department of Physical Medicine and Rehabilitation, Vanderbilt University Medical Center, Ste 1318, 2201 Childrens Way, Nashville, TN, 37212, USA; e Mayo Clinic Libraries, Mayo Clinic, 200 First St SW, Rochester, MN, 55905, USA; f Department of Physical Medicine and Rehabilitation, University of Utah School of Medicine, 590 Wakara Way, Salt Lake City, UT, 84108, USA; g Department of Pain Medicine, Mayo Clinic, 4500 San Pablo Rd, Jacksonville, FL, 32224, USA

**Keywords:** Chronic knee pain, Total knee arthroplasty, Genicular nerve, Radiofrequency, Systematic review

## Abstract

**Objective:**

Summarize the therapeutic pain-reducing effects of GnRF for refractory post-TKA knee pain. A secondary objective was to summarize improvements in physical function after GnRF.

**Methods:**

A protocol was registered, and a database search conducted by an experienced librarian of all available studies in the English language up until November 3, 2021. Study inclusion criteria were randomized controlled trials (RCTs), prospective and retrospective longitudinal studies, cross-sectional studies, case series, case reports, studies involving adults ≥18 years of age, and studies written about the use of GnRF for the alleviation of chronic knee pain after receiving a TKA. The study quality and risk of bias was assessed using NHLBI Study Quality of Assessment Tools and Murad et al.'s Quality Assessment of Case Reports. Certainty in the evidence was assessed using the Grading of Recommendations, Assessment, Development, and Evaluation approach.

**Results:**

A total of 229 studies were screened, 11 met the inclusion criteria, and 265 patients underwent GnRF. Study designs included 1 double-blind pragmatic RCT, 5 retrospective cohort studies, 2 retrospective case series, and 3 case reports. The overall study quality assessment demonstrated three studies had “good”, six “fair”, and two “poor” quality. There have been positive responses to GnRF for post-TKA chronic knee pain in a range of 30–100% of patients.

**Conclusions:**

According to GRADE, there is limited evidence, associated with low certainty to support the use of GnRF to ameliorate chronic knee pain after TKA, largely due to inconsistency and risk of bias. The studies included in this review reported positive results in pain and disability, and relatively few adverse events.

## Abbreviations

GnRF: genicular nerve radiofrequency treatments
Gn-tRFA: traditional radiofrequency ablation
Gn-cRFA: cooled radiofrequency ablation
Gn-PRF: pulsed radiofrequency neuromodulation
TKA: total knee arthroplasty
SMGN: superomedial genicular nerve
SLGN: superolateral genicular nerve
IMGN: inferomedial genicular nerve
ILGN: inferolateral genicular nerve
NVM: nerve to vastus medialis
NVL: nerve to vastus lateralis
NVI: nerve to vastus intermedius
RFN: recurrent fibular nerve
IPBSN: infrapatellar branch of the saphenous nerve
RCT: randomized controlled trials
NRS: numerical rating pain score
OKS: Oxford Knee Score
KSS: Knee Society Score
GPE: global perceived effect
VAS: visual analog pain score
PDI: pain disability index
WOMAC: Western Ontario and McMaster Universities Osteoarthritis score

## 1 Introduction

Severe symptomatic knee osteoarthritis is a common degenerative condition that affects 37% of adults over the age of 60, most frequently presenting as unilateral or bilateral knee pain, decreased function, limited joint mobility, and eventual disability [[1], [2], [3]]. The traditional treatment of choice for symptomatic knee osteoarthritis that has failed conservative management, including activity modification, physical therapy, medications, and injections, is total knee arthroplasty (TKA) [4]. This surgical intervention has a high rate of success for most patients with chronic knee pain secondary to symptomatic osteoarthritis [4], however, approximately 15% continue to suffer from chronic knee pain postoperatively which may be more severe than the pain due to osteoarthritis in the native knee [[5], [6], [7]]. After ruling out mechanical or infectious etiologies, persistent pain may remain unexplained or be considered neuropathic [6,8].

The most common first-line treatments for chronic post-TKA pain include topical and non-invasive measures, gabapentinoid anticonvulsants, and antidepressants [9]. Historically, many patients were prescribed opioid medications to treat severe refractory postoperative knee pain [7,10,11]; approximately 30% continued to refill their postoperative opioid prescriptions for more than 3 months, and often up to 12 months after surgery due to ongoing knee pain [12,13]. Due to the risk for tolerance, dependence, addiction, and development of central sensitization and hyperalgesia, long-term opioid use should rarely be considered [9,14].

A promising set of interventions have emerged with the potential to alleviate chronic post-TKA pain: genicular nerve radiofrequency treatments (GnRF). There have been mainly two types of radiofrequency studied for the genicular nerves: pulsed and continuous. Pulsed radiofrequency involves isolated activity at the electrode tip but delivers a lower temperature with an alternation of repeating pulses, or bursts of heat, and silence, causing non-ablative nerve disruption at a cellular level with minimal damage [[15], [16], [17]]. Continuous radiofrequency heats tissue at a percutaneous electrode tip by enacting molecular oscillation, friction generation, and heat-related coagulation of neurons at the target site [15,16]. This second type of radiofrequency can be further characterized into two subtypes: traditional and cooled radiofrequency ablation. The traditional subtype is performed at 80 ​°C and creates an elliptical-shaped lesion, while the cooled subtype is performed using a water-cooled probe at 60 ​°C, and creates a larger, spherical-shaped lesion, providing a more expansive area of denervation [18].

The genicular nerves supply sensory innervation to the anterior knee capsule and follow an anatomical pattern that can be treated via percutaneous intervention [19]. In 2011, Choi et al. published the first randomized controlled trial (RCT) of GnRF, targeting the superomedial (SMGN), superolateral (SLGN), and inferomedial (IMGN) genicular nerves [20]. Multiple anatomic and clinical studies have considered the appropriate targets for ablation of the genicular nerves for knee pain, with emerging evidence suggesting that the traditional 3–4 needle ablation may not adequately capture the intended targets. A number of cadaveric studies have suggested additional and varied sensory innervation of the anterior capsule of the knee [21,22]. A 14-study review by Roberts et al. demonstrated that, in addition to SMGN, SLGN, and IMGN, there were 7–8 other nerves that innervate the anterior knee joint capsule, including inferolateral genicular nerve (ILGN), common fibular nerve (CFN), recurrent fibular nerve (RFN), infrapatellar branch of the saphenous nerve (IPBSN), nerve to vastus medialis (NVM), nerve to vastus lateralis (NVL) and nerve to vastus intermedius (NVI), including both the medial and lateral branches [23]. In addition, the posterior knee joint capsule innervation can penetrate anteriorly into the infrapatellar fat pad and the current GnRF protocols do not target the posterior joint innervation, including the popliteal plexus, articular branches of the tibial nerve, posterior branch of the obturator nerve, and posterior branch of CFN or sciatic nerve [23]. This illustrated that simple adjustments to GnRF target sites could provide a more broadly comprehensive intervention, considering the complex innervation of the knee capsule, in both native and post-TKA knees [[21], [22], [23]].

Here, we present a systematic review whose primary objective is to summarize the therapeutic pain-reducing effects of GnRF for refractory post-TKA knee pain. A secondary objective was to summarize improvements in physical function after GnRF.

## 2 Methods

### 2.1 Study protocol

Preferred reporting items for systematic reviews and meta-analysis (PRISMA) guidelines were followed when performing this systematic review [24]. The protocol was registered in the International Prospective Register for Systematic Reviews database (CRD42021284611) on November 11, 2021 [25]. An amendment to this protocol was submitted on January 1, 2022, to specify that we excluded conference abstracts.

### 2.2 Search strategy

A comprehensive search of several databases from each database's inception to November 3, 2021, English language, was conducted. The databases included Ovid MEDLINE(R) and Epub Ahead of Print, In-Process & Other Non-Indexed Citations, and Daily, Ovid EMBASE, Ovid Cochrane Central Register of Controlled Trials, Ovid Cochrane Database of Systematic Reviews, and Scopus. The search strategy was designed and conducted by an experienced librarian with input from the study's principal investigator. Controlled vocabulary supplemented with keywords was used to search for genicular nerve radiofrequency ablation for knee pain in adults. The actual strategy listing all search terms used and how they are combined is available in Appendix A.

### 2.3 Study-selection process

We included studies written about the use of GnRF for the alleviation of chronic knee pain after receiving a TKA including RCTs, prospective and retrospective longitudinal studies, cross-sectional studies, case series, case reports, and studies involving adults ≥18 years of age. Exclusion criteria included conference abstracts (included in Appendix B: Notable Excluded Studies) [[26], [27], [28], [29], [30], [31], [32], [33]], reviews, and studies regarding perioperative genicular nerve blocks, blocks without subsequent GnRF, blocks of other peripheral extremity nerves, radiofrequency treatments to non-knee joints, or blocks for osteoarthritis without a previous TKA. No restrictions were imposed regarding image guidance method or radiofrequency parameters.

In the first phase of review, four independent reviewers screened all the titles and abstracts identified by the search strategy which was provided by an expert librarian. In the second review phase, four independent reviewers then screened the full texts of all studies procured by the first phase. Disagreements were resolved by consensus. The references of the included articles were also used to screen for other potentially relevant articles for inclusion.

### 2.4 Data extraction

Data were extracted by six independent reviewers using a templated electronic database. Each article was individually assessed by two reviewers. Disagreements were resolved by consensus. Extracted data comprised authorship, publication year, study design, sample size, patient demographics, prognostic block status, radiofrequency procedure and details, follow-up, outcome measures to assess post-GnRF pain and functional improvement, and adverse events.

### 2.5 Risk of bias and methodological quality assessment

Risk of bias was assessed by seven independent reviewers using the National Heart, Lung, and Blood Institute (NHLBI) Study Quality Assessment Tools [34] for controlled intervention studies, observational studies, and case series, as well as the Quality Assessment of Case Reports according to Murad et al. [35] Each article was individually assessed by three reviewers.

### 2.6 Certainty in the evidence

Certainty in the evidence was assessed using the Grading of Recommendations Assessment, Development, and Evaluation (GRADE) approach (J.B.M.), which was adapted for use with quantitative data that are not combinable in meta-analysis [36,37].

### 2.7 Evidence synthesis

Due to the clinical heterogeneity in the retrieved study characteristics, a meta-analysis was not performed. The summary of our findings is presented using a narrative approach, which is indicated when the key clinical factors varied, and when the content studied used dissimilar methods of execution [38,39]. A narrative approach for evidence synthesis has been previously used to study patients with variable pain syndromes [40,41].

## 3 Results

### 3.1 Characteristics of included studies

**Fig. 1:**
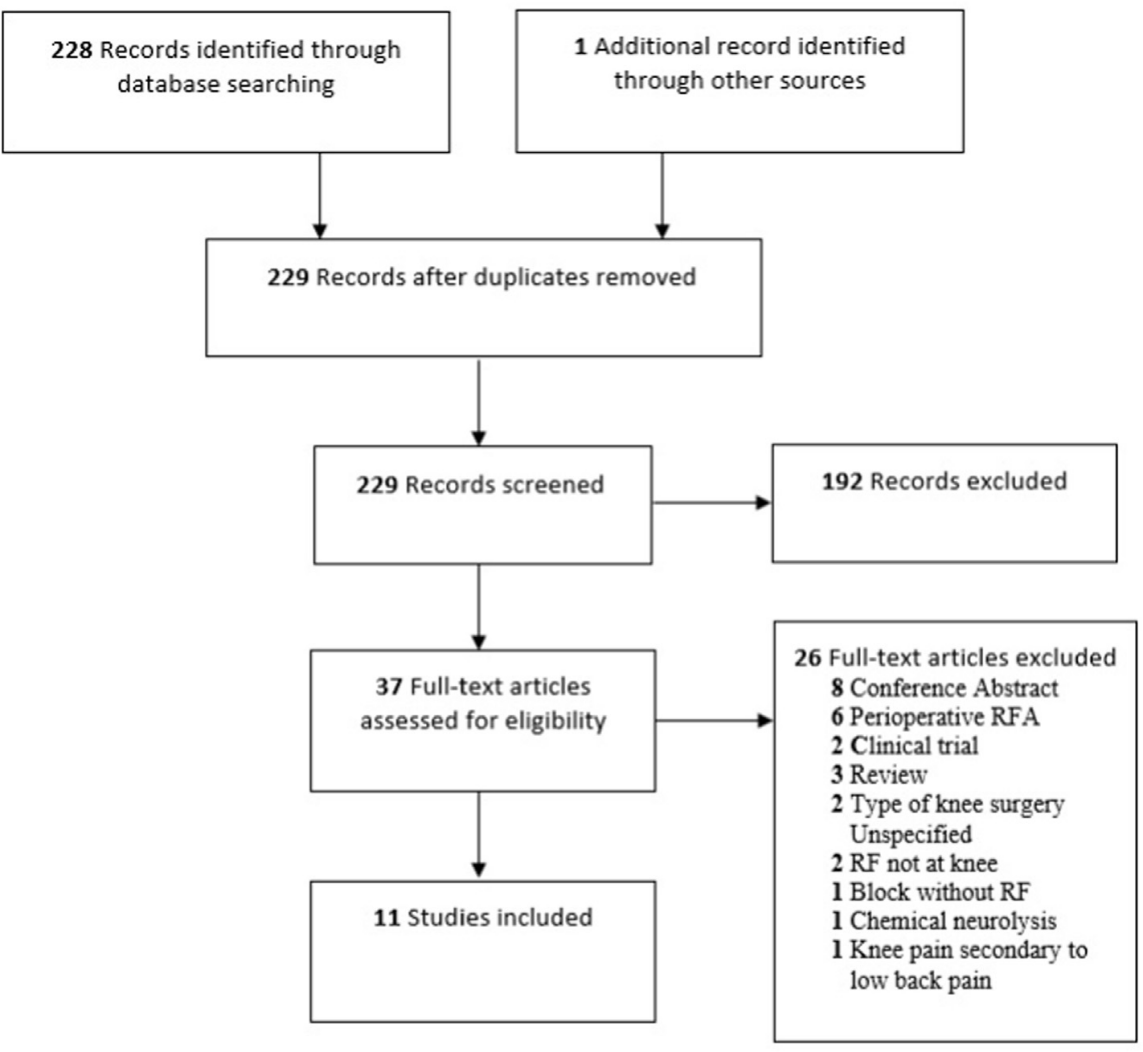
Preferred reporting items for systematic reviews and meta-analyses (PRISMA) flowchart of the study selection process.

A flow diagram of the study selection process is shown in Fig. 1. Eleven studies met criteria for inclusion in the review, including 265 patients [4,[42], [43], [44], [45], [46], [47], [48], [49], [50], [51]]. Patient characteristics are included in Table 1. Study designs included 1 double-blind pragmatic RCT, 5 retrospective cohort studies, 2 retrospective case series, and 3 case reports. These studies included the following types of GnRF: traditional radiofrequency ablation (Gn-tRFA), cooled radiofrequency ablation (Gn-cRFA), pulsed radiofrequency neuromodulation (Gn-PRF), and 3-tined radiofrequency ablation (Gn-3t-RFA) of the genicular nerves. Study characteristics and parameters of radiofrequency treatments are shown in Table 2.

**Table 1:** Patient stratification from included studies.

Included Studies	Mean Age (years)	Male/Female (%)	Mean BMI (kg/m^2^)
Qudsi-Sinclair (2017)	67.4 (±7.2)	29/71	NR
Belba (2021)^a^	62.2 (±16.9)	32/68	NR
Baber (2020)	62.8 (±3.4)	40/60	NR
Protzman (2014)	48	100/0	NR
Sylvester (2017)	68	0/100	NR
Kapural (2019)^a^	61	43/57	34
Eshraghi (2021)	66.1 (±11.2)	37/63	34.5 (±10.1)
Menzies (2015)	68	100/0	48.82
Erdem (2019)	78 (±2.9)	33/67	26.3 (±2.8)
Chen (2021)^a^	64.3 (±15.2)	36/64	NR
Koshi (2020)^a^	55 [median]	91/9	NR

BMI ​= ​body mass index; NR ​= ​not recorded.

a Patient data was reported as a whole and not subdivided into native knee osteoarthritis and post-total knee arthroplasty patients.

**Table 2:** Study characteristics and parameters of radiofrequency treatments.

Included Studies	Design	n	Targeted Nerves	Diagnostic Block	Radiofrequency Details	Outcome Measures
**Traditional Radiofrequency Ablation of the Genicular Nerves (Gn-tRFA)**						
Qudsi-Sinclair (2017)	Double-blind pragmatic randomized controlled trial	14	SMGN SLGN IMGN	NR	Temperature: 80 ​°C Time: 90 ​s Cannula: 22 ​G, 10 ​mm	NRS OKS KSS Overall improvement perception
Belba (2021)	Retrospective Cohort	43	SMGN SLGN IMGN	2 ​ml 1% Lidocaine	Temperature: 70 ​°C Time: 90 ​s Cannula: 21 ​G, 5 ​mm	NRS, GPE
Baber (2020)	Retrospective Case Series	5	SMGN SLGN IMGN	1 ​ml 1% Lidocaine	Temperature: 85 ​°C Time: 90 ​s Repetitions: 3 Cannula: 20 ​G, 15 ​mm	% subjective improvement
Protzman (2014)	Case Report	1	SMGN SLGN IMGN	1 ​ml 1% Bupivacaine & 1 ​ml 1% Lidocaine	Temperature: 80 ​°C Time: 90 ​s Cannula: NR	VAS
Sylvester (2017)	Case Report	1	SMGN SLGN IMGN	1 ​ml 0.5% Bupivacaine	Temperature: 80 ​°C Time: 120 ​s Cannula: NR	% subjective improvement
Chen (2021)^a^	Retrospective Cohort	140	SMGN SLGN IMGN ILGN NVM NVL NVI RFN IPBSN	0.5–2 ​ml 0.5% Bupivacaine	Temperature: 80–90 ​°C Time: 75–150 ​s Cannula: 18–20 ​G, 10 ​mm	NRS
**Cooled Radiofrequency Ablation of the Genicular Nerves (Gn-cRFA)**						
Kapural (2019)	Retrospective Cohort	21	SMGN SLGN IMGN	1 ​ml Bupivacaine	Temperature: 60 ​°C Time: 150 ​s Cannula: 17 ​G, 4 ​mm	VAS
Eshraghi (2021)	Retrospective Cohort	31	SMGN SLGN IMGN	NR	Temperature: 60 ​°C Time: 150 ​s Cannula: 17 ​G	NRS PDI
Menzies (2015)	Case Report	1	SMGN SLGN IMGN	NR	Temperature: 70 ​°C Time: 90 ​s Cannula: NR	% subjective improvement OKS
Chen (2021)^a^	Retrospective Cohort	140	SMGN SLGN IMGN ILGN NVM NVL NVI RVN IPBSN	0.5–2 ​ml 0.5% Bupivacaine	Temperature: 60 ​°C Time: 150 ​s Cannula: 17 ​G, 4 ​mm	NRS
**Pulsed Radiofrequency of the Genicular Nerves (Gn-PRF)**						
Erdem (2019)	Retrospective Cohort	6	SMGN SLGN IMGN	NR	Temperature: 42 ​°C Time: 120 ​s Repetitions: 3 Cannula: 22 ​G, 5 ​mm	VAS WOMAC
Chen (2021)^a^	Retrospective Cohort	140	SMGN SLGN IMGN ILGN NVM NVL NVI RVN IPBSN	0.5–2 ​ml 0.5% Bupivacaine	Temperature: 42 ​°C Time: 120 ​s Pulse duration: 20 ​ms Repetitions: 1-2 Cannula: 18–20 ​G, 10 ​mm	NRS
**Three-tined Radiofrequency Ablation of the Genicular Nerves (Gn-3t-RFA)**						
Koshi (2020)	Retrospective Case Series	2	SMGN SLGN IMGN NVM NVL NVI	NR	Temperature: 80 ​°C Time: 120 ​s Cannula: 18 ​G, 5 ​mm, 3-tined	NRS

SMGN ​= ​superomedial genicular nerve; SLGN ​= ​superolateral genicular nerve; IMGN ​= ​inferomedial genicular nerve; ILGN ​= ​inferolateral genicular nerve; NVM ​= ​nerve to vastus medialis; NVL ​= ​nerve to vastus lateralis; NVI ​= ​nerve to vastus intermedius; RFN ​= ​recurrent fibular nerve; IPBSN ​= ​infrapatellar branch of the saphenous nerve; NR ​= ​not recorded; NRS ​= ​numerical rating pain score; OKS = Oxford Knee Score; KSS = Knee Society Score; GPE ​= ​global perceived effect; VAS ​= ​visual analog pain score; PDI ​= ​pain disability index; WOMAC = Western Ontario and McMaster Universities Osteoarthritis score.

a Chen (2021) is listed under 3 categories because it utilized different types of radiofrequency treatments. Each with an “∗” beside it indicates that it is the same study.

### 3.2 Traditional radiofrequency ablation of the genicular nerves (Gn-tRFA)

Qudsi-Sinclair et al. [50] performed a double-blind pragmatic RCT of 28 patients comparing Gn-tRFA (n ​= ​14) versus genicular nerve injection combining local anesthetic with corticosteroid (AC) (n ​= ​14) for post-TKA knee pain. Inclusion criteria for this study included patients older than 18 years and greater than 6 months of post-TKA knee pain. The 3 target sites were identified using fluoroscopic guidance at the medial and lateral diaphyseal-metaphyseal femoral transition points (for SMGN and SLGN, respectively), as well as the medial diaphyseal-metaphyseal tibial transition point (for the IMGN). None of the 28 patients received a prognostic genicular nerve block. Fourteen patients received a Gn-tRFA to the SMGN, SLGN, and IMGN using a 22-gauge, 10 ​mm active tip radiofrequency cannula for 90 ​s at 80 ​°C. The Gn-tRFA group demonstrated a significant improvement from baseline to months 6 and 12 in mean numeric rating pain scores (NRS) (2.6 [±2.7], *P* ​< ​0.001; 2.1 [±2.2], *P* ​< ​0.001), OKS (7.53[±10.56], *P* ​< ​0.01; 7.86[±12.54], *P* ​< ​0.01), and Knee Society Score (KSS) (7.93[±15.18], *P* ​< ​0.01; 13.67[±18.98], *P* ​< ​0.01). The AC group also demonstrated significant improvements from baseline to months 6 and 12 in mean NRS (0.9 [±2.87], *P* ​< ​0.001; 0.15 [±2.64], *P* ​< ​0.001), OKS (11.87 [±4.35], *P* ​< ​0.01; 9.62 [±9.45], *P* ​< ​0.01), and KSS (19.25 [±15.63], *P* ​< ​0.01; 17.62 [±13.11], *P* ​< ​0.01). Between group differences for NRS, OKS, and KSS were not significant (*P ​>* 0.05). Regarding overall perception of pain improvement, 65% (95% CI, 35–87%) of the Gn-tRFA group reported they were “very much better” or “much better” compared to only 35% (95% CI, 13–65%) of the AC group after 6 months, with 43% (95% CI, 18–71%) compared to 21% (95% CI, 5–51%) after 1 year. There were no reported complications, although the patients did report pain when the radiofrequency cannula touched periosteum. The study was likely insufficiently powered to detect an intergroup difference given that both groups used an active interventional treatment, and the total sample size of patients who had Gn-tRFA was only 14 patients.

Belba et al. [43] performed a retrospective cohort study of 46 patients with persistent postsurgical knee pain (PPSP), of which 43 (93%) had a prior TKA and 15 (33%) had a revision TKA, who underwent an ultrasound-guided Gn-tRFA. The average baseline numerical rating score (NRS) was 7.3. Each patient first underwent a diagnostic block using 1 ​mL of 2% lidocaine at each of the SMGN, SLGN, and IMGN sites. If the diagnostic block demonstrated at least 50% pain reduction, lesioning was then performed using a 21-gauge traditional RF cannula with a 5 ​mm active tip for 90 ​s at 70 ​°C. At week 6, the mean NRS was 6.1 with NRS reduction of >50% in 9/46 patients (19.6%, 95% CI, 9–34%) and global perceived effect (GPE) ​> ​50% in 15/46 (32.6%, 95% CI, 20–48%). At the second timepoint, the mean NRS was 5.5 and GPE was >50% in 4/13 (30.8%, 95% CI, 9–65%) patients, with a subjective increase in functionality in 6/13 (46.2%, 95% CI, 19–75%). This was the only study in this systematic review that reported any post-procedure complications. Six patients had adverse events, including hypoesthesia, instability while walking, increase in pain, self-limiting hematoma, flare of CRPS; one patient had a severe adverse event with development of new CRPS.

Baber et al. [42] presented a retrospective case series of 8 cases, including 5 with prior TKA, who underwent Gn-tRFA. Prior to the ablative procedure, each received prognostic blocks using 1 ​mL of 1% lidocaine at the SMGN, SLGN, and IMGN using the landmarks of the medial and lateral junctions at the distal femoral shaft and epicondyles and at the medial junction of the proximal tibia and epicondyle, respectively. If >50% pain reduction was achieved, a confirmatory block using 1 ​mL of 0.25% bupivacaine was performed in the same locations. If >50% pain reduction was achieved from this second block, the patients proceeded to Gn-tRFA with a 20-gauge traditional RF cannula with a 15 ​mm curved active tip at 85 ​°C for 90 ​s with 3 totals repetitions at each site. In this TKA group, 40% (95% CI, 9–76%) of patients had >50% pain relief at week 3, 40% (95% CI, 9–76%) had >80% pain relief at month 3, and 20% (95% CI, 3–65%) had 100% pain relief at rest and 66% (95% CI, 24–91) pain relief with ambulation at month 5. There were no complications reported.

Protzman et al. [49] presented a case report of a 48-year-old male with post-TKA chronic knee pain who received Gn-tRFA lesioning at the SMGN, SLGN, and IMGN for 90 ​s at 80 ​°C. The RF cannula active tip length was not reported. The patient reported a visual analog pain score (VAS) of 0 ​at ​week 2 and month 3 follow-up. In this post-treatment window, a physical therapist noted gains in both strength and range of motion, with additional improvement in ambulation up and down the stairs without using a handrail. There were no complications reported.

Sylvester and Goree [51] presented a case report of a 68-year-old female with post-TKA chronic radiating posterior thigh and knee pain who received Gn-tRFA lesioning at the SMGN, SLGN, and IMGN for 120 ​s at 80 ​°C. The RF cannula active tip length was not reported. The patient reported complete alleviation of the pain at month 3 follow-up, except for mild (2/10) pain that returned nightly after her normal daily activities. There were no complications noted.

### 3.3 Cooled radiofrequency ablation of the genicular nerves (Gn-cRFA)

Kapural et al. [46] conducted a retrospective cohort study that included 183 patients with knee pain who received Gn-cRFA, 21 of whom had a history of TKA. After a successful trial of 1 ​ml bupivacaine genicular blocks, lesioning was performed at the SMGN, SLGN, and IMGN using a 17-gauge radiofrequency cannula with a 4 ​mm active tip and generator settings of 150 ​s at 60 ​°C (intralesional temperature exceeding 80 ​°C). One hundred and nineteen patients reported >50% pain relief and 141 had ≥2 visual analog pain scores (VAS) point decrease after Gn-cRFA. The average VAS for all 183 patients was 8.5 ​at baseline and 4.2 after Gn-cRFA. Although the authors stated that follow-up would occur at month 3 and month 6 after Gn-cRFA, they did not report when the previously mentioned pain outcomes were assessed. While data for the sub-cohort with a history of TKA was not included, the authors reported no significant difference in VAS improvement between the TKA and non-TKA groups (*P* ​= ​0.542), indicating clinical effectiveness of Gn-cRFA for post-TKA knee pain. There were no reported complications. Specific sites for lesioning were not provided in this study.

Eshraghi et al. [45] performed a retrospective cohort study of 219 patients with knee pain who received Gn-cRFA, including 31 who had a prior TKA. Lesioning was performed at the SMGN, SLGN, and IMGN at the junction between the femoral or tibial shaft and the epicondyle, respectively, at least 50% across the diaphysis, using a 17-gauge radiofrequency and generator settings of 60 ​°C for 150 ​s. The RF cannula active tip length and the performance of prognostic blocks were not reported. Lesioning was followed by 1 ​cc of solution containing 3 ​cc bupivacaine 0.25% and 40 ​mg triamcinolone acetate injected through each needle. Median follow-up was 169 days. While data for the sub-cohort with a history of TKA was not included, 93% (95% CI, 89–96%) of patients had pain relief assessed by NRS, with a decrease in mean NRS after Gn-cRFA (2.8, 95% CI [−3 to −2], *P* ​< ​0.001). Of note, the morphine equivalent dose did not significantly change for 80.7% (95% CI, 75–86%) of patients. 67.3% (95% CI, 60–73%) of patients demonstrated a decrease in pain disability index (PDI) scores, with a mean reduction of 31.5% (*P* ​< ​0.0001). Ultimately, the authors found that Gn-cRFA improved pain relief without reduction of opioid consumption. There were no reported complications.

Menzies and Hawkins [48] presented a case report of a 68-year-old male with bilateral post-TKA chronic knee pain who received Gn-cRFA at the SMGN, SLGN, and IMGN for 90 ​s at 70 ​°C, with only one lesion per site. The RF cannula active tip length was not reported and a progostnic block was not performed prior to Gn-cRFA. The patient anecdotally did report marked OKS improvements for both knees and improved quality of life, minimal knee pain, less reliance on analgesics, and ability to walk more freely after cooled radiofrequency ablation.

### 3.4 Pulsed radiofrequency of the genicular nerves (Gn-PRF)

Erdem and Sir [4] conducted a retrospective cohort study of 23 patients with knee pain who received Gn-PRF, including 6 with a history of TKA. For the purpose of this systematic review, we re-categorized this retrospective cohort study as a 6-patient case series, only including the patients with a history of TKA. Prior to the procedure, they identified each of the 3 target sites using ultrasound guidance, locating the SMGN at the medial femoral shaft, SLGN at the lateral femoral shaft, and the IMGN as it advanced around the tibial neck and medial epicondyle. Each of the six patients with a history of TKA underwent Gn-PRF using a 22-gauge radiofrequency cannula with a 5 ​mm active tip for 120 ​s at 42 ​°C for 3 consecutive cycles at the SMGN, SLGN, and IMGN. The voltage output, frequency, and pulse width were not reported. A prognostic block was not performed prior to Gn-PRF. Four patients (67%, 95% CI, 22–96%) reported a >50% reduction in visual analog scale (VAS) pain score at week 3 and month 3 follow-up. A significant reduction in mean post-Gn-PRF VAS pain scores and Western Ontario and McMaster Universities Osteoarthritis (WOMAC) scores were observed from baseline to week 3 and month 3 follow-up (VAS: 4, *P* ​< ​0.01; 3.8, *P* ​< ​0.01; WOMAC: 24.4, *P* ​< ​0.01; 18.9, *P* ​< ​0.01). In this small case series, Gn-PRF generally appeared to have a favorable effect. There were no reported complications.

### 3.5 A combination of Gn-tRFA, Gn-cRFA, and Gn-PRF

Chen et al. [44] performed a retrospective cohort study of 265 patients with knee pain who received GnRF, including 140 (52.9%) with a prior TKA. These GnRF procedures were performed using the typical anatomical protocol at the SMGN, SLGN, and IMGN, but were adjusted to an expanded anatomical protocol depending on pain location, patient tolerance, and physician preference, which included the targeting of the ILGN, NVM, NVL, NVI, RFN, and/or IPBSN. Prior to GnRF, prognostic blocks were performed using 0.5–2 ​ml of 0.5% bupivacaine at each predetermined site. One of three types of GnRF was then performed, including Gn-tRFA, Gn-cRFA, or Gn-PRF. Gn-tRFA was performed at 80–90 ​°C for 75–150 ​s with 18–20-gauge radiofrequency cannulas with 10 ​mm active tips, Gn-cRFA was performed at 60 ​°C for 150 ​s (intralesional temperature exceeding 80 ​°C) with 17-gauge radiofrequency cannula with 4 ​mm active tips, and Gn-PRF was performed at 42 ​°C with voltage output 40–60 ​V, 2 ​Hz frequency, 20 ​ms pulses in 1 ​s cycle, and 120 ​s duration per cycle with 1–2 cycles per target site with 18–20-gauge radiofrequency cannulas with 10 ​mm active tips. Of the 140 post-TKA patients, 88 (63%, 95% CI, 54–71%)) had a positive outcome, defined as ​≥ ​30% pain relief, assessed by NRS, lasting at least 3 months, without additional intervening interventions. In the regression analysis, the therapeutic response of GnRF was not significantly different between native and post-TKA knees (native ​= ​79.1%, post-TKA ​= ​63.0%, *P* ​= ​0.09), however, many of their patients were treated with large lesions, multiple burns, and ablation of additional nerves. There were no reported complications.

### 3.6 Three-tined radiofrequency ablation of the genicular nerves (Gn-3t-RFA)

Koshi et al. [47] conducted a retrospective case series of 11 patients with knee pain who received Gn-3t-RFA, including 2 (18.2%) with a prior TKA. Prognostic blocks were not performed. This procedure was performed using an 18-gauge three-tined RFA cannula with 5 ​mm active tips (Diros RF Trident) at 80 ​°C for 120 ​s. This cannula creates a pyramidal lesion with the largest lesion diameter closest to the cannula's distal tips. This study used an expanded protocol targeting the SMGN, SLGN, IMGN, NVM, NVL, and NVI. The 2 patients with prior TKA reported 95% and 75% pain relief at month 1 post-procedure, respectively, as well as 80% and 70% pain relief at month 6 post-procedure, respectively, assessed by NRS. There were no reported complications.

### 3.7 Risk of bias and methodological quality assessment

**Fig. 2:**
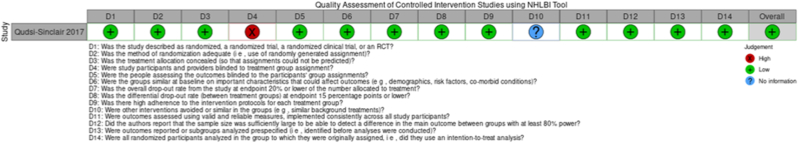
Quality Assessment of Controlled Intervention Studies using NHLBI Tool.

**Fig. 3:**
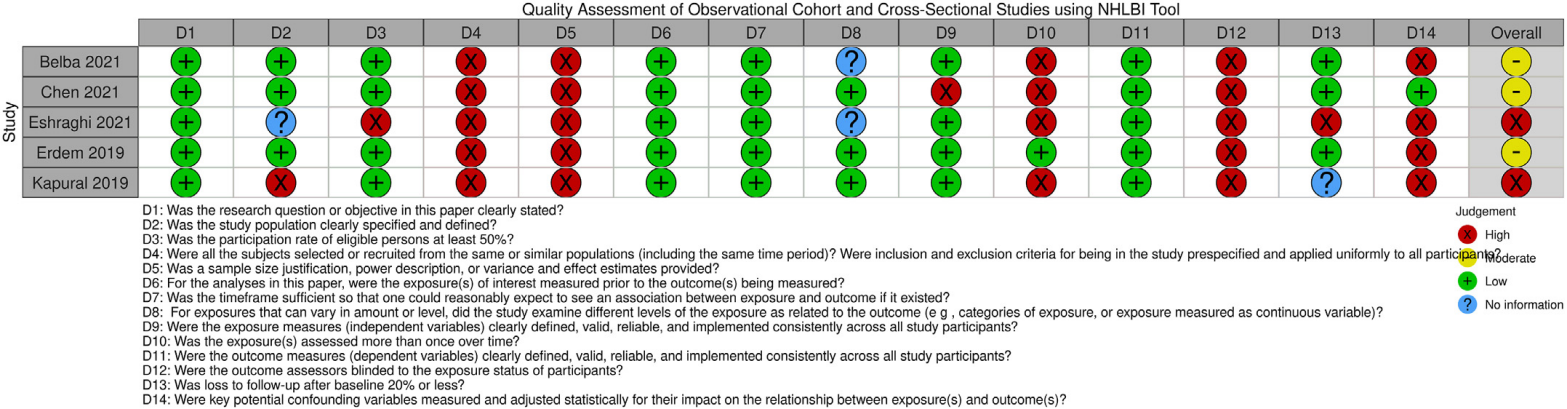
Quality Assessment of Observational Cohort and Cross-Sectional Studies using NHLBI Tool.

**Fig. 4:**
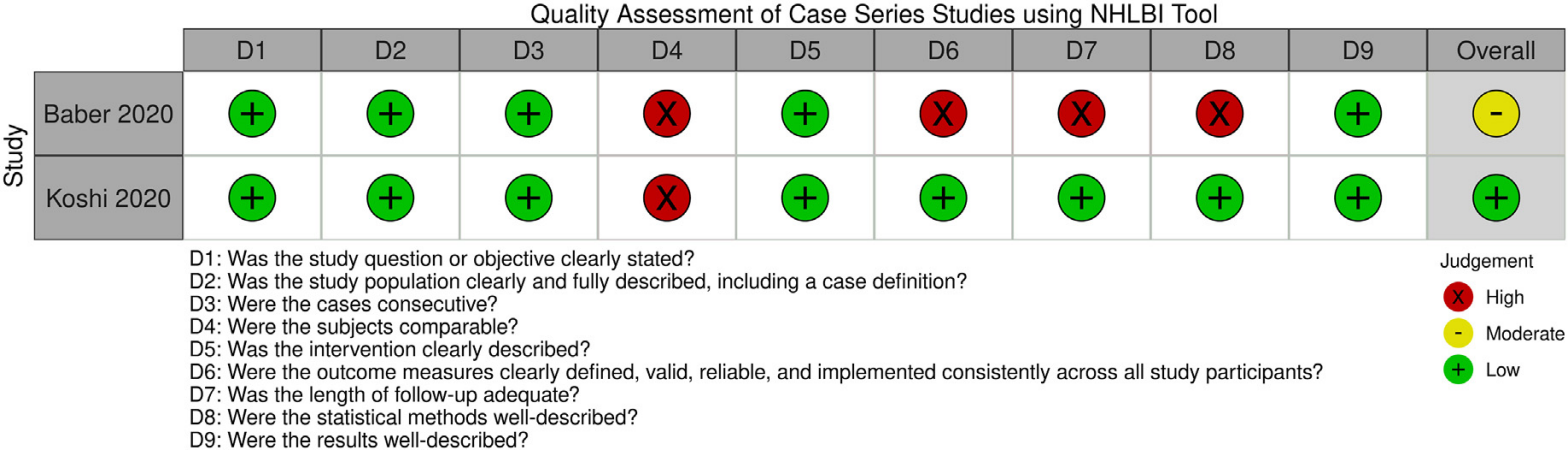
Quality Assessment of Case Series Studies using NHLBI Tool.

**Fig. 5:**
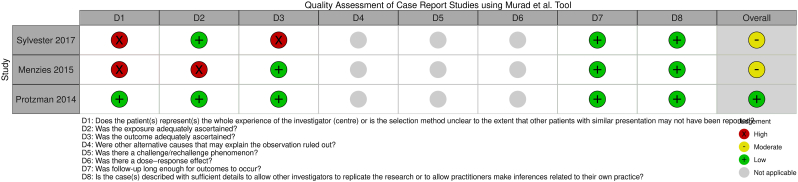
Quality Assessment of Case Report Studies using Murad et al. Tool.

The overall study quality assessment demonstrated three studies had “good” quality [47,49,50], six had “fair” quality [4,[42], [43], [44],^48^,^51^], and two had “poor” quality [45,46] (Fig. 2, Fig. 3, Fig. 4, Fig. 5) [52]. These ratings can be stratified by radiofrequency type. Of note, one study used multiple radiofrequency types and has been included in three assessments below [44].

In the Gn-tRFA studies, two studies were assessed as “good” [49,50] and four “fair” [[42], [43], [44],^51^] study quality for heterogeneity of treatment type outcomes [44], small sample size [42,51], inadequate outcome ascertainment [51], inadequate follow-up time period [42], and lack of confounding variable assessment [43]. The one RCT was assessed as having “good” study quality and “low” risk of bias, with the critiques being the lack of discussion on how the sham RFA was performed in the steroid group, lack of blinding by those performing the procedures, and being underpowered [50]. In the Gn-cRFA studies, two studies were assessed as “fair” [44,48] study quality for inadequate exposure ascertainment [48], small sample size [48], and heterogeneity of treatment type outcomes [44], and two “poor” [45,46] study quality for inadequate patient population definition [45,46], lack of blinding [45,46], and lack of confounding variable assessment [45,46]. In the Gn-PRF studies, two studies demonstrated “fair” [4,44] study quality for heterogeneity in patient population [4,44], heterogeneity of treatment type outcomes [44], small sample size [4], and lack of confounding variable assessment [4]. The one Gn-3t-RFA study demonstrated “good” study quality [47].

### 3.8 Certainty in the evidence

The GRADE assessment indicated that certainty in the evidence was “low” to support the use of GnRF to ameliorate chronic knee pain after TKA. These assessments were made due to inconsistency and risk of bias. The certainty in the evidence was the same for both pain and function.

## 4 Discussion

The key findings of this systematic review include (1) there are currently 3 different types of GnRF performed for post-TKA chronic knee pain, including Gn-tRFA, Gn-cRFA, and Gn-PRF; (2) typically the targeted nerves include the SMGN, SLGN, and IMGN, although there are protocols that expand the number of nerve targets past these original three; and (3) there have been positive responses to GnRF for post-TKA chronic knee pain in one small RCT and ten uncontrolled case series and studies. We observed considerable heterogeneity across studies in terms of lesion sites, complicating our ability to draw clear comparisons regarding the efficacy of Gn-tRFA, Gn-cRFA, and Gn-PRF for post-TKA pain.

The IPBSN primarily provides cutaneous innervation and may, in a small subset of individuals, additionally innervate the joint capsule at the superior portion of the inferomedial quadrant or the anteromedial part of the capsule [22,23]. Chen et al. was the only study identified in this systematic review that specifically targeted the IPBSN [44]. Due to the small innervating coverage, it may not be necessary to target the IPBSN for chronic native knee pain but may be an important target, and significant pain generator, in post-TKA knee pain if the nerve sustained periprocedural damage. Neuralgia over the front of the knee, which correlates with periprocedural transection of the IPBSN and sometimes neuroma formation [[53], [54], [55]], is an occasional occurrence after TKA, resulting in stiffness and post-TKA pain which can be treated with targeted intervention at the IPBSN. The viability of this intervention can be identified by a diagnostic block to determine if ablation of the IPBSN should be pursued [23].

A recent publication by Chen et al. investigated the clinical and technical factors associated with RFA outcomes which provides further considerations when designing a GnRF protocol for post-TKA knee pain [44]. Targeting more than three nerves, including the addition of RFN, NVM, NVL, IPBSN and NVI, had better outcomes in patients with both native and non-native knees [44]. A recent 11-patient case series by Koshi et al. utilized a 7-lesion approach to the SMGN, SLGN, IMGN, NVM, NVI, and NVL using a three-tined electrode in which the 91% of the patients reported >50% improvement of knee pain at month 1 follow-up [47,56,57]. This strengthens the notion that an increased number of targets may be warranted for a more complete pain relief protocol. Post-TKA chronic knee pain can be more challenging to treat than osteoarthritis in native knees. Targeting additional genicular nerves may be more important in this population than those with native knee osteoarthritis to enhance the chances of achieving a meaningful outcome.

Our review identified few reported adverse events [43]. While the included studies on GnRF for chronic knee pain after TKA have shown promising benefits without any serious adverse events, there have been reported rare complications of GnRF in native knee osteoarthritis, including iatrogenic hematoma [58], skin burns [59], pes anserine tendon injury [60], and even septic arthritis [61]. Large cohort and/or registry work is needed to confirm the safety of genicular nerve interventions for recalcitrant chronic knee pain after TKA, though extrapolation from studies of genicular nerve radiofrequency ablation in patients with pain in a native knee indicate a favorable safety profile. A key problem noted by Mazor et al*.* is that patients who suspect that an adverse event has occurred in their care do not report this to the treating physician [62]. Furthermore, physicians who learn about adverse events may not report them because of a lack of training, culture of blame, or fear of medicolegal repercussions [[63], [64], [65]]. The lack of long-term follow-up limits the ability to capture potential adverse events as well as a reliable assessment of clinical effectiveness of the procedure.

Traditionally the ILGN and RFN are avoided during the standard protocol for GnRF due to its proximity to the fibular neck and common peroneal nerve, which, if injured, carries the subsequent risk of foot drop. However, emerging studies have laid out protocols to safely target the RFN, in addition to other nerves, that may contribute to knee pain [[66], [67], [68]]. Chen et al. specifically targeted both the ILGN and RFN and reported no complications [44].

A diagnostic genicular nerve block is often utilized prior to GnRF with the thought that if the patient receives good pain relief from the block that they will receive a similar relief from the subsequent GnRF [[42], [43], [44],^46^,^49^,^51^]. While this has not been specifically tested in a post-TKA pain population, McCormick et al. conducted a prospective randomized trial of prognostic genicular nerve blocks to determine the predictive value for outcomes of Gn-cRFA in native knee osteoarthritis. Seventeen (58.6%) and 16 (64%) of patients in the prognostic and no block groups, respectively, had ≥50% pain relief at month 6 (*P* ​= ​0.34). They concluded that prognostic genicular nerve blocks did not predict an improved the rate of Gn-cRFA treatment success [69]. In this systematic review there was heterogeneity regarding the use of prognostic genicular nerve blocks with their utilization in only 6/11 (54.5%) of the included studies [[42], [43], [44],^46^,^49^,^51^].

There is a paucity of published evidence regarding the use of GnRF for post-TKA chronic knee pain. There are no prospective, double-blinded, placebo-controlled RCTs studying this specification indication, which is needed to establish efficacy. However, there are two completed, yet-to-be published clinical trials evaluating the effect of genicular radiofrequency ablation for the relief of chronic post-arthroplasty knee pain [70,71]. This systematic review also highlights the heterogeneity of radiofrequency treatment parameters (see Table 2) and future research with a standardized approach to GnRF in the post-TKA population would be beneficial.

### 4.1 Limitations

Although we endeavored to provide a rigorous qualitative assessment of the available data regarding pain and functional outcomes following GnRF for chronic post-TKA knee pain, our study has several limitations. We excluded conference abstracts which does subject our review to publication bias. Given the highly technical nature of this topic we wanted to restrict our analysis to studies had been subject to peer review. Although we searched the Ovid databases for clinical trials, we did not conduct a formal search of clinicaltrials.gov for additional ongoing clinical trials that have yet to be published, as this was outside of the scope of our review to analyze pain and functional outcomes following GnRF for post-TKA pain. By only including studies published in English we may have missed studies that would have been pertinent to our review.

## 5 Conclusion

According to GRADE, there is low certainty in the evidence to support the use of GnRF to ameliorate chronic knee pain after TKA due to inconsistency and risk of bias. This systematic review was limited by relatively few (n ​= ​11), primarily nonrandomized studies (n ​= ​10) pertaining to GnRF of post-TKA knees, with many of these studies (n ​= ​7) including fewer than 20 patients who undergo GnRF. In addition, a majority (n ​= ​8) were assessed as having less than “good” study quality. However, the studies included in this review reported positive results in pain and disability ranging 30–100%, and relatively few adverse events. There is a need for continued study of GnRF for chronic knee pain after TKA. Sham-controlled RCTs are needed to establish efficacy of GnRF in this population and large prospective trials are needed to clarify whether GnRF is effective for the treatment of post-TKA pain. The present review indicates a need to standardize lesion targets for GnRF in order to be able to establish efficacy of this procedure for post-TKA pain, whether anatomical changes following surgery should be considered when performing this procedure in patients after knee replacement, and whether the use of ultrasound might confer additional benefit in post-surgical patients when hardware may obscure the ability to identify cannulae depth as a way to target common genicular nerve locations.

## Funding source

This research did not receive any specific grant from funding agencies in the public, commercial, or not-for-profit sectors.

## Declaration of competing interest

The authors declare the following financial interests/personal relationships which may be considered as potential competing interests: Zachary L. McCormick, MD, serves on the Board of Directors of the Spine Intervention Society.

## Figures and Tables

**Table:** 

#	Searches	Results
1	exp Arthroplasty, Replacement, Knee/	49001
2	exp Knee Joint/	138158
3	exp Knee/	85135
4	exp Osteoarthritis, Knee/	65537
5	(knee or knees).ti,ab,kw.	407022
6	1 or 2 or 3 or 4 or 5	442236
7	exp Pain/	1921477
8	exp Pain Management/	224442
9	(arthralgia∗ or neuralgia∗ or pain∗ or polyarthralgia∗).ti,ab,kw.	2111514
10	7 or 8 or 9	3010252
11	6 and 10	118738
12	exp Radiofrequency Ablation/	75321
13	exp Pulsed Radiofrequency Treatment/	1367
14	(((Radiofrequency or "Radio-frequency" or "Transvenous Electric∗" or "*Trans*-venous Electric∗") and (Ablat∗ or treat∗ or therap∗ or block∗)) or "catheter ablation" or RFA).ti,ab,kw.	117570
15	12 or 13 or 14	143534
16	(genicular or geniculate).ti,ab,kw.	20576
17	15 and 16	382
18	11 and 17	359
19	limit 18 to ("all adult (19 plus years)" or "young adult (19–24 years)" or "adult (19–44 years)" or "young adult and adult (19–24 and 19–44)" or "middle age (45–64 years)" or "middle aged (45 plus years)" or "all aged (65 and over)" or "aged (80 and over)") [Limit not valid in CCTR, CDSR,Embase; records were retained]	309
20	limit 19 to (adult <18 to 64 years> ​or aged <65+ years>) [Limit not valid in CCTR, CDSR, Ovid MEDLINE(R), Ovid MEDLINE(R) Daily Update, Ovid MEDLINE(R) PubMed not MEDLINE, Ovid MEDLINE(R) In-Process,Ovid MEDLINE(R) Publisher; records were retained]	253
21	limit 18 to ("all infant (birth to 23 months)" or "all child (0–18 years)" or "newborn infant (birth to 1 month)" or "infant (1–23 months)" or "preschool child (2–5 years)" or "child (6–12 years)" or "adolescent (13–18 years)") [Limit not valid in CCTR, CDSR,Embase; records were retained]	275
22	limit 21 to (embryo or infant or child or preschool child <1 to 6 years> ​or school child <7 to 12 years> ​or adolescent <13 to 17 years>) [Limit not valid in CCTR, CDSR, Ovid MEDLINE(R), Ovid MEDLINE(R) Daily Update, Ovid MEDLINE(R) PubMed not MEDLINE, Ovid MEDLINE(R) In-Process,Ovid MEDLINE(R) Publisher; records were retained]	90
23	22 not 20	0
24	18 not 23	359
25	limit 24 to english language [Limit not valid in CDSR; records were retained]	313
26	limit 24 to no language specified [Limit not valid in CDSR; records were retained]	38
27	25 or 26	351
28	limit 27 to (editorial or erratum or note or addresses or autobiography or bibliography or biography or blogs or comment or dictionary or directory or interactive tutorial or interview or lectures or legal cases or legislation or news or newspaper article or overall or patient education handout or periodical index or portraits or published erratum or video-audio media or webcasts) [Limit not valid in CCTR, CDSR,Embase, Ovid MEDLINE(R), Ovid MEDLINE(R) Daily Update, Ovid MEDLINE(R) PubMed not MEDLINE, Ovid MEDLINE(R) In-Process,Ovid MEDLINE(R) Publisher; records were retained]	5
29	27 not 28	346
30	remove duplicates from 29	237

**Table:** 

Author (year)	Title	Reason for exclusion
Thomas (2020) [32]	Is There a Role for Cooled Radio-Frequency Ablation in the Management of Persistently Painful Total and Revision Knee Arthroplasty? Our Early Experience.	Conference abstract
Yoshimura (2019) [33]	Efficacy of ultrasound-guided radiofrequency ablation for genicular nerve in patients with chronic knee pain after total knee arthroplasty.	Conference abstract
Hintz (2018) [29]	Knee genicular nerve block and radiofrequency ablation for the treatment of knee osteoarthritis: A retrospective chart review of outcomes for both pre-and post-TKA patients.	Conference abstract
Akin Takmaz (2018) [26]	Effectiveness of genicular nerves pulsed radio frequency ablation for treatment of persistent chronic pain after knee arthroplasty.	Conference abstract
Mirdamada (2015) [31]	Genicular neurotomy as an alternative option for chronic knee pain after total knee arthroplasty: A case report.	Conference abstract
Gong (2015) [28]	Ultrasound-guided genicular nerve block for persistent knee pain after knee surgery.	Conference abstract
Ivanova-Stoilova (2014) [30]	Radiofrequency denervation of genicular nerves for treatment of intractable knee pain (CLINICAL SERIES).	Conference abstract
Danko (2013) [27]	Radiofrequency ablation of genicular nerves for the treatment of chronic knee osteoarthritis.	Conference abstract

## Appendix A Actual Search Strategies

### Ovid

Database(s): EBM Reviews - Cochrane Central Register of Controlled Trials September 2021, EBM Reviews - Cochrane Database of Systematic Reviews 2005 to October 27, 2021, Embase 1974 to 2021 November 02, Ovid MEDLINE(R) and Epub Ahead of Print, In-Process, In-Data-Review & Other Non-Indexed Citations and Daily 1946 to November 02, 2021.

Search Strategy:
